# ICTV Virus Taxonomy Profile: Spinareoviridae 2022

**DOI:** 10.1099/jgv.0.001781

**Published:** 2022-11-17

**Authors:** Jelle Matthijnssens, Houssam Attoui, Krisztián Bányai, Corina P. D. Brussaard, Pranav Danthi, Mariana del Vas, Terence S. Dermody, Roy Duncan, Qín Fāng (方勤), Reimar Johne, Peter P. C. Mertens, Fauziah Mohd Jaafar, John T. Patton, Takahide Sasaya (笹谷孝英), Nobuhiro Suzuki (鈴木信弘), Taiyun Wei (魏太云)

**Affiliations:** 1 University of Leuven, Leuven, Belgium; 2 National Institute for Agricultural Research (INRA), Maisons Alfort, France; 3 Veterinary Medical Research Institute, Budapest, H-1143, Hungary; 4 NIOZ Royal Netherlands Institute for Sea Research & University of Utrecht, Texel, The Netherlands; 5 Indiana University, Bloomington, USA; 6 Instituto de Agrobiotecnología y Biología Molecular (IABIMO), Buenos Aires, Argentina; 7 University of Pittsburgh School of Medicine, Pittsburgh, Pennsylvania, USA; 8 Dalhousie University, Halifax, Nova Scotia, Canada; 9 Wuhan Institute of Virology, Wuhan, PR China; 10 German Federal Institute for Risk Assessment, Berlin, Germany; 11 University of Nottingham, Leicestershire, UK; 12 Ecole Nationale Vétérinaire d’Alfort, Maisons Alfort, France; 13 National Agriculture and Food Research Organization, Fukuyama, Japan; 14 Okayama University, Kurashiki, Japan; 15 Fujian Agriculture and Forestry University, Fuzhou, PR China

**Keywords:** ICTV report, taxonomy, *Spinareoviridae*, *Reovirales*

## Abstract

*Spinareoviridae* is a large family of icosahedral viruses that are usually regarded as non-enveloped with segmented (9–12 linear segments) dsRNA genomes of 23–29 kbp. Spinareovirids have a broad host range, infecting animals, fungi and plants. Some have important pathogenic potential for humans (e.g. Colorado tick fever virus), livestock (e.g. avian orthoreoviruses), fish (e.g. aquareoviruses) and plants (e.g. rice ragged stunt virus and rice black streaked dwarf virus). This is a summary of the ICTV Report on the family *Spinareoviridae*, which is available at ictv.global/report/spinareoviridae.

## Virion

**Fig. 1.**
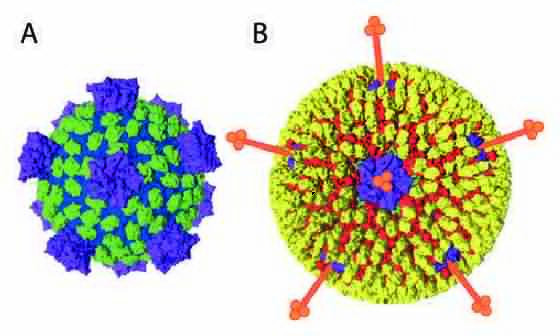
Spinareovirid particle structure coloured according to functional similarity (blue – core shell, green – middle layer, yellow – outer capsid, purple – RNA capping, red – membrane penetration, orange – receptor binding) (shown schematically). (a) Subviral particle. (b) Virion. The diameter of the mature virion is approximately 80 nm (excluding spikes). Adapted from [7].

Spinareovirid particles are icosahedral (Table 1). The protein capsid is organized as 1–3 concentric layers of capsid proteins, with an overall diameter of 50–85 nm [1]. Members of the family *Spinareoviridae* have spikes or turrets at the 12 icosahedral vertices of the subviral particle (Fig. 1), in contrast to members of the family *Sedoreoviridae*, which have an almost spherical or ‘smooth’ appearance.

**Table 1.** Characteristics of members of the family *Spinareoviridae*

Example:	mammalian orthoreovirus-3 Dearing (T3D; HM159613–22), species *Mammalian orthoreovirus*, genus *Orthoreovirus*
Virion	Non-enveloped, icosahedral, 60–85 nm virions composed of 1–3 concentric capsid protein layers
Genome	23–29 kbp of segmented linear dsRNA, with each of the 9–12 segments ranging from 0.5 to 4.8 kbp
Replication	Replication occurs in the cytoplasm in electron-dense structures called viroplasms, viral inclusions or viral factories
Translation	From full-length transcribed mRNAs that possess a 5′-terminal cap but no poly(A)-tail
Host range	Mammals, aquatic animals (fish, mammals, crustaceans, molluscs), birds, reptiles, arthropods, fungi and plants
Taxonomy	Realm *Riboviria,* kingdom *Orthornavirae*, phylum *Duplornaviricota,* class *Resentoviricetes,* order *Reovirales*: >8 genera and >55 species

## Genome

Spinareovirids contain 9–12 segments of linear dsRNA comprising 23–29 kbp in total, with individual segments ranging from 0.5 to 4.8 kbp. The positive-sense strands of each duplex are modified with a 5′-terminal type 1 cap structure but no 3′-poly(A) tail. The viral RNAs are mostly monocistronic with relatively short 5′- and 3′-non-coding regions, although some segments have a second or third functional ORF [2,3].

## Replication

Virus entry into cells varies between genera but usually results in loss of outer-capsid components. The resulting transcriptionally active particles are released into the cytoplasm. The 5′-capped mRNAs are synthesized by structural enzymatic components of the viral particle and released into the cytoplasm through pores at the five-fold icosahedral vertices of the virion. Viral inclusions, also known as viral factories, are distributed throughout the cytoplasm. These neo-organelles are sites of viral mRNA synthesis, genome replication and particle assembly [4]. Sets of a single copy of each capped mRNA are incorporated into progeny virus particles [5]. These mRNAs serve as templates for negative-strand synthesis, thereby reconstituting genomic encapsidated dsRNAs. Progeny virions are released without compromising cell viability (e.g. budding) or following cell lysis, depending on the cell type [6].

## Taxonomy

**Fig. 2.**
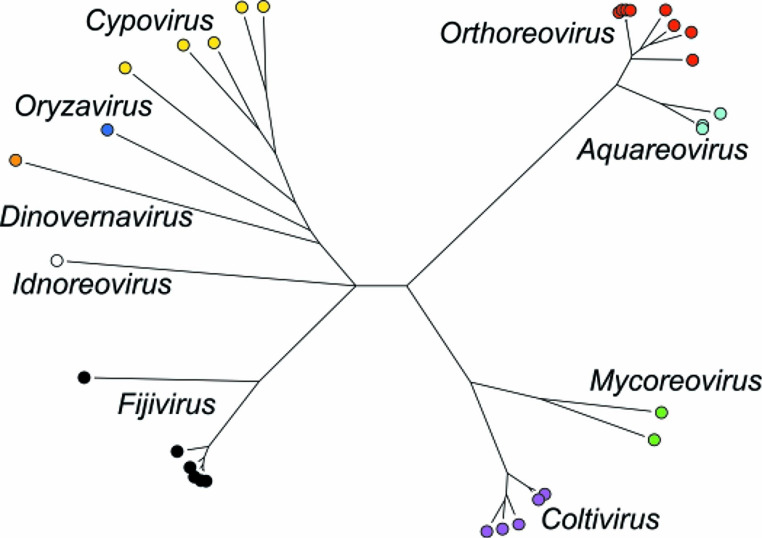
Spinareovirus phylogeny based on RNA-directed RNA polymerase amino acid sequences. For details see online Report.

Current taxonomy: ictv.global/taxonomy. The family *Spinareoviridae* includes multiple genera (Fig. 2) and >55 species of viruses infecting mammals, aquatic animals (fish, mammals, crustaceans, molluscs), birds, reptiles, arthropods, fungi and plants. The number of genome segments (9–12) is characteristic of viruses within a single genus, although members of the genus *Mycoreovirus* can have either 11 or 12 segments. Other factors distinguishing different genera are host (and vector) range, disease signs and capsid structure. The amino acid sequence of the relatively conserved RNA-directed RNA polymerase can be used for comparison across taxonomic boundaries. Among members of a species, protein and RNA sequences are relatively conserved, being serologically cross-reactive and including specific RNA packaging signals. This high degree of functional and structural compatibility allows viable progeny virus strains to be generated by reassortment between viruses of the same species.

## Resources

Full ICTV Report on the family *Spinareoviridae*: ictv.global/report/spinareoviridae
